# Management of chronic pain with chronic opioid therapy in patients with substance use disorders

**DOI:** 10.1186/1940-0640-8-21

**Published:** 2013-12-16

**Authors:** Yu-Ping Chang, Peggy Compton

**Affiliations:** 1 University of Buffalo School of Nursing, Buffalo, 3435 Main Street Wende Hall 201E, Buffalo, NY 14221, USA; 2 School of Nursing and Health Studies, Georgetown University, Washington, DC, USA

**Keywords:** Chronic pain, Chronic opioid therapy, Addiction/substance use disorder, Relapse prevention

## Abstract

Substance use disorders (SUDs), whether active or in remission, are often encountered in patients with chronic nonmalignant pain. Clinicians are challenged when managing chronic pain while facing substance abuse issues during the course of chronic opioid therapy (COT). Further, the interrelated behavioral symptomatology of addiction and chronic pain suggests that if one disorder is untreated, effective treatment of the other in not possible. Incomplete understanding of the overlapping presentations of the two disorders, coupled with insufficient management of both conditions, leads to undertreated pain and premature discharge of SUD patients from pain treatment. In order to achieve pain relief and optimal functionality, both conditions need to be carefully managed. This paper reviews the prevalence of SUDs in chronic pain patents; the overlapping presentation of the two disorders; risk factors and stratification for addiction; identification of addiction in the chronic pain population; and suggestions for treating patients with COT, with an emphasis on relapse prevention. With appropriate assessment and treatment, COT for chronic pain patients with a history of SUD can be successful, leading to improved functionality and quality of life.

## Introduction

Treating chronic pain with chronic opioid therapy (COT) in individuals with a history of a substance use disorder (SUD), whether active or in remission, presents a challenge to pain clinicians. This is, in part, due to concerns about the patient relapsing to active substance abuse in the course of COT, as analgesic treatment enables and legitimizes drug use for patients with SUDs [1-3]. In addition, clinicians may confuse “drug-seeking” behaviors with addictive disease, resulting in poor treatment outcomes such as premature discharge of patients from pain care [4]. Misconceptions persist as chronic pain patients with SUDs are often treated by clinicians who have insufficient training in addiction, and evidence-based clinical guidelines for managing pain while addressing SUDs are lacking [2,5]. The goal of chronic pain treatment in patients with SUDs is the same as that for patients without SUDs: specifically, to maximize functionality while providing pain relief. However, reluctance to prescribe opioids and poor understanding of the complex relationship between pain and addiction too often result in undertreated pain in this population [6].

A review of the literature reveals that no empirical studies have been conducted to investigate the risks and benefits associated with COT in chronic pain patients with a history of SUD [7]. This paper reviews what is known about the prevalence of SUDs in chronic pain patents; links between pain and addiction; risk factors and stratification for addiction and implications for COT; and indicators of addiction in this population. Suggestions for treating chronic pain in SUD patients receiving COT are outlined with an emphasis on the role of relapse prevention in successful outcomes.

### Prevalence of SUDs in chronic pain patients

In attempting to estimate the prevalence or presence of SUD in chronic pain patients, terminology becomes important (Table 1). It is increasingly understood that SUD cannot be defined by *physical dependence* and *tolerance*, as these are predictable physiologic consequences of chronic opioid use. Reflecting this, in the *Diagnostic and Statistical Manual of Mental Disorders,* 5th Edition (DSM-V), tolerance and withdrawal are not counted as criteria for the substance use and addictive disorder diagnosis if a patient is taking an opioid analgesic under medical supervision [8].

**Table 1:** Definition of terminology

**Terms**	**Definitions**
Misuse	Taking a prescription for a reason or at a dose or frequency other than for which it was prescribed; this may or may not reflect POUD*.
	Use of a medication for nonmedical use, or for reasons other than prescribed. For example, altering dosing or sharing medicines, which has harmful or potentially harmful consequences. It does not refer to use for mind-altering purposes [9].
Abuse	Misuse with consequences. The use of a substance to modify or control mood or state of mind in a manner that is illegal or harmful to oneself or others. Potentially harmful consequences include accidents or injuries, blackouts, legal problems, and sexual behavior that increases the risk of human immunodeficiency virus infection [9].
Physical dependence	A state of adaptation manifested by a drug class-specific withdrawal syndrome that can be produced by abrupt cessation, rapid dose reduction, decreasing blood level of the drug, and/or administration of an antagonist [10].
Tolerance	A state of adaptation in which exposure to a drug induces changes that result in a diminution of one or more opioid effects over time [10].
Addiction	A primary, chronic, neurobiologic disease with genetic, psychosocial, and environmental factors influencing its development and manifestations. It is characterized by behaviors that include one or more of the following: impaired control over drug use, compulsive use, continued use despite harm, and craving [10].
Pseudo-addiction	An iatrogenic syndrome of “addiction-like” behaviors in which the patient seeks opioids to relieve pain—such as seeking different doctors, self-adjusting the opioid dose, early refills of opioids, etc.—rather than to achieve pleasure or other nonpain-related effect [11]. At times mistaken for true addiction, these behaviors tend to resolve and function improves once analgesia is better addressed. Further defined as “behavioral changes in patients that seem similar to those in patients with opioid dependence or addiction but are secondary to inadequate pain control” [12].
Therapeutic dependence	Drug-seeking secondary to anxiety about having an adequate supply of medication [13].
Opioid-induced hyperalgesia	A state of nociceptive sensitization caused by exposure to opioids. The condition is characterized by a paradoxical response, whereby a patient receiving opioids for the treatment of pain could actually become more sensitive to certain painful stimuli. The type of pain experienced might be the same as the underlying pain or might be different from the original underlying pain [14,15].
Aberrant drug-related behavior	Taking a controlled substance medication in a manner that is not prescribed; causes for this may include:
	• lack of understanding about how to take the opioid appropriately
	• external pressures, such as to give to another person for his or her pain
	• chemical coping
	• pseudoaddiction (see below), including:
	– physical tolerance and resultant inadequate pain control
	– opioid-resistant pain
	– opioid-induced hyperalgesia
	– progression of their pain generator or disease
	• addiction or substance use disorder (such as POUD)
	• diversion
	A behavior outside the boundaries of the agreed-on treatment plan which is established as early as possible in the doctor-patient relationship [16].

*POUD: prescription opioid use disorder.

Albeit using imperfect indicators, it has been estimated that the prevalence of opioid abuse in chronic pain patients ranges between 20-24% across health-care settings [17]. Using a survey approach and DSM-IV criteria, Boscarino and colleagues [18] completed phone interviews with a random sample of 705 chronic pain patients receiving COT in primary care and specialty pain treatment. They found that 26% of those reported a current opioid use disorder and 36% had a life-time opioid use disorder, findings that were replicated using DSM-V criteria [19]. A systematic review of literature synthesizing 21 studies published prior to February 2012 showed that the overall prevalence of current SUDs in chronic pain patients ranges from 3% to 48% depending on the population sampled [7]. The lifetime prevalence of any SUD ranged from 16% to 74% in patients visiting the emergency department, with those visiting for opioid refill having the highest rate. Further, it has been reported that 3.3% to 11.5% of chronic pain patients with a history of SUD may develop opioid addiction or abuse, whereas only 0.19% to 0.59% of those without a prior or current history of SUD develop the same [20].

### Syndrome of pain and addiction

Chronic pain and addiction are best conceptualized as a syndrome. In some individuals with addictive disease, pain is identified as a factor contributing to their addiction. It has been hypothesized that untreated pain may be a risk factor for relapse for individuals with addiction in remission [21]; however, it has also been suggested that exposure to opioids in chronic pain patients with a history of SUD puts them at risk for opioid abuse and/or relapse [22].

Physiological and psychological aspects of active addictive disease can make pain more difficult to treat. Chronic use of opioid drugs appears to affect the processing of pain stimuli through sympathetic stimulation, hypothalamic-pituitary-adrenal axis dysregulation, and proinflammatory immune-system activation, resulting in increased sensitivity to pain or decreased pain tolerance [14,23]. These responses suggest that the presence of both chronic pain and opioid addiction may result in a reorganization of nociceptive pathways in the brain that subsequently cause increased pain perception, or so-called opioid-induced hyperalgesia.

Savage and Schofferman [24] described a “syndrome of pain facilitation” occurring in patients with untreated addiction and pain, such that the pain experience is worsened by the presence of addiction. Individuals who abuse alcohol, cocaine, opioids, or other drugs often experience alternating withdrawal and intoxication due to unstable blood levels of drug. Similarly, for individuals receiving opioids, withdrawal can activate the sympathetic nervous system, with concomitant muscle tension, irritability, and dysphoria, further contributing to discomfort.

Mood, sleep, and personality disorders can aggravate pain symptoms and are frequently comorbid in patients with chronic pain [25-30]. The literature indicates that chronic pain patients with untreated depression respond poorly to pain treatment [31,32]. Due to functional limitations, chronic pain patients may become isolated and unable to engage in physical and social activities, which further contribute to the severity of the chronic pain experience [33,34]. Unable to fulfill work and domestic roles, they are also likely to experience interpersonal conflicts, financial difficulty, and poor social support, all of which are detrimental to adequate chronic pain management [35].

Similarly, mood disorders, including depression and anxiety, are common sources of distress in patients with SUD [36-38], which likewise diminish patient functionality [33]. The overall dysfunction associated with addiction contributes to distress and disability. Further, pain patients with active addition are unlikely to comply with nonopioid pain treatment regimens, including physical therapy and behavioral interventions. The signs and sequelae of untreated addiction thwart improvement with COT.

Unresolved emotional and social distress coupled with persistent pain may lead patients to self-medicate these uncomfortable feeling states with opioids. When self-medication becomes a coping mechanism, substance use can progress to a disorder, or cause relapse in patients with a history of SUD. In a recent study of 1334 patients receiving COT for noncancer chronic pain, those with moderate and severe depression were more likely to self-medicate nonpain symptoms with prescription opioids and to misuse their prescription opioid by self-increasing doses than were those without depression [39].

### Risk factors and risk stratification for addiction in pain patients receiving COT

Clinicians should conduct a comprehensive risk assessment for opioid abuse or misuse when considering use of COT. The assessment should include known risk factors for addiction, including a personal or family history of substance abuse, childhood adverse events (eg, physical or sexual abuse, childhood neglect), psychiatric symptoms, and functional impairment (pain disability, sleep disturbance). With respect to pain symptoms, assessment in patients considered at risk for addictive disease must include careful delineation of the nociceptive and affective components of the pain syndrome; identification of associated factors that perpetuate pain; and identification of pain-related risk factors for opioid abuse and relapse. Degree of functionality (Table 2) in the presence of chronic pain is a critical assessment, as the effectiveness of COT is evident in this domain.

**Table 2:** **Evidence of functional restoration **[8]

	
•	Physical capabilities
•	Psychological intactness
•	Satisfying family and social interactions
•	Appropriate health-care utilization
•	Appropriate medication use

Risk stratification approaches are indicated for selecting chronic pain patients for COT, and those with a history of SUD are considered at high risk for poor treatment response [40]. Being a chronic disease, it is critical to ensure that SUD’s continue to be addressed while treating chronic pain. Gourlay, Heit, and Almahrezi [41] propose a 10-step universal precaution approach as a minimum standard of care for all chronic pain patients receiving COT (Table 3). This model of universal precautions is framed within a biopsychosocial approach and designed to reduce stigma, improve outcomes, and decrease risks associated with COT pain management for all patients [41], regardless of SUD history.

**Table 3:** Ten steps of universal precautions

	
1.	Make a diagnosis with appropriate differential.
2.	Perform a psychological assessment, including risk of addictive disorders.
3.	Obtain informed consent.
4.	Use a treatment agreement.
5.	Conduct assessment of pain level and function before and after the intervention.
6.	Begin an appropriate trial of opioid therapy with or without adjunctive medications and therapies.
7.	Reassess pain score and level of function.
8.	Regularly assess the 4 “As” of pain medication: Analgesia, Activity, Adverse effects, and Aberrant behavior.
9.	Periodically review pain diagnosis and co-occurring conditions, including addictive disorders.
10.	Document initial evaluation and follow visits.

Adopted from Gourlay DL et al [41].

#### Risk factors

The risk factors for opioid abuse, misuse, or other aberrant drug-related behaviors in chronic pain patients receiving COT have been well-described, with a prior history of opioid abuse being the best predictor for both current and lifetime opioid use disorder in chronic pain patients [18,42]. Other important but less consistent risk factors for opioid abuse include pain-related functional limitations/impairments (including sleep disturbances); current cigarette smoking; a family history of substance abuse; a history of a mood disorder (eg, current post-traumatic disorder or depression); history of child sexual abuse or child neglect; involvement in the legal system; and significant psychosocial stressors [43-45]. Demographic correlates of opioid misuse in this patient population include age, gender, ethnicity, and employment status. Previous studies indicate that younger chronic pain patients (under age 65) are at higher risk for opioid abuse [18,46]. With respect to gender, women with chronic pain who reported more emotional issues and affective distress were at increased risk for opioid misuse, whereas men with legal problems tended to predict misuse prescription opioids [47].

Boscarino and colleagues [18] found that the chance of opioid abuse increases if a chronic pain patient has multiple risk factors, such that the odds ratio (OR) of a current opioid use disorder in chronic pain patients who present with four predictors (age, depression, psychotropic medication, and pain impairment) is 8.01. If the patient also has a history of severe opioid dependence and abuse, the risk of current opioid use disorder increased dramatically (OR, 56.36).

#### Risk stratification and monitoring strategies

**Figure 1:**
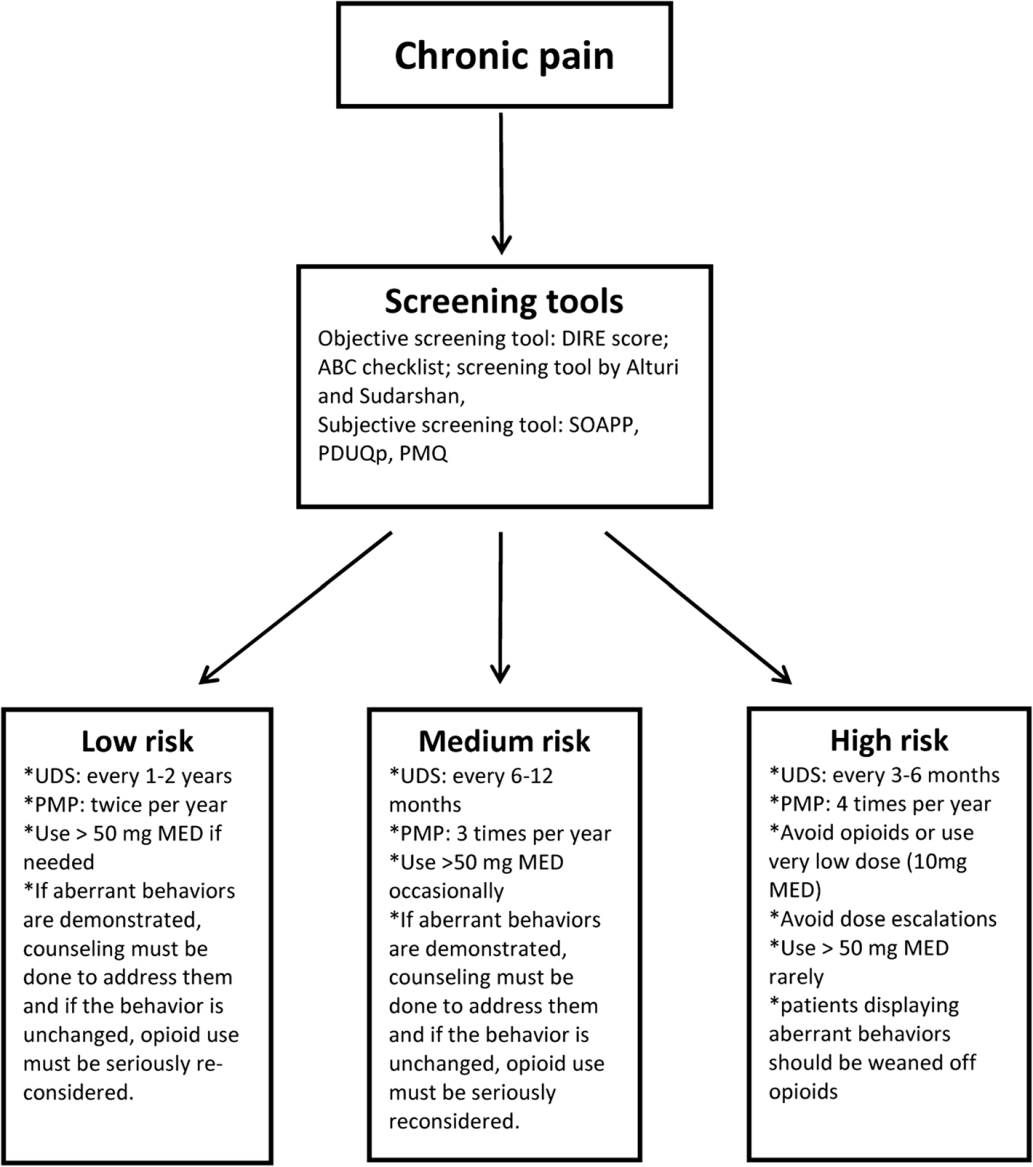
**Stratification of chronic pain patients by use of screening tools (cited in text) into high, medium, and low risk groups for opioid abuse, monitoring patients by using urine dug screening (UDS), Prescription Monitoring Programs (PMPs) and aberrant behaviors; and lastly establishing suggested dose (MED: Morphine Equivalent dose) limits.** Adopted from [48].

Atluri and colleagues [48] have suggested an algorithmic approach to prevent opioid abuse in chronic pain treatment by stratifying patients into high-, medium-, and low-risk groups using one of several validated screening tools (Figure 1). These tools include subjective questionnaires, eg, Screener and Opioid Assessment for Patients with Pain (SOAPP) [49], Pain Medication Questionnaire (PMQ) [50], and Prescription Drug Use Questionnaire Patient Version (PUDQP) [51]); and objective tools, eg, Addiction Behavior Checklist (ABC) [52], Diagnosis, Intractability, Risk, Efficacy (DIRE) [53], and Current Opioid Misuse Measure (COMM) [54].

Based on the stratification of risk, different approaches are suggested. Individuals with a history of SUD are categorized as high risk, thus, frequent monitoring of medication use, urine drug testing (UDT) every three to six months, and reviewing Prescription Monitoring Program (PMP) reports every two to four months are recommended. Although not uniformly supported in the literature, these authors suggest that opioids should be avoided or prescribed only in low doses; a >50 mg morphine-equivalent dose should be used only rarely and only in specialized settings.

Building on the universal precautions, management can be tailored to the care for patients at risk for SUD. For example, in addition to the general components written in the opioid treatment agreement or contract, the clinician should stipulate that participation in ongoing addiction treatment (eg, 12-step meetings, outpatient treatment, or individual counseling/therapy) be required for COT prescription. More frequent office visits are required to better assess opioid use behaviors, opioid efficacy, and signs of relapse. Clinicians should prescribe opioids to these patients in smaller amounts, without refills, and conduct pill counts at each visit. If appropriate, a family member or a close friend can be included in the treatment plan (for example, to dispense medications).

Clinicians should collect urine samples more frequently for mass spectrometry confirmatory toxicology screen [55]. In a large prospective study of chronic pain patients receiving COT (N = 500), Manchikanti and colleagues [56] found significant reductions in overall illicit drug use with adherence-monitoring procedures combined with random UDT. Continued monitoring using UDT significantly decreased the incidence of illicit drug use over time [57]. It is important to note that, although UDT is an objective measure of the presence of drugs and their metabolites, it is not a stand-alone indicator of adherence or addiction; thus, the results should be openly discussed with patients along with assessment of other indicators of relapse. False-positive and false-negative results can occur with UDT, so with unexpected findings, toxicology analyses should be verified and/or repeated.

Brief cognitive-behavioral interventions have been shown to reduce the risk of COT misuse in chronic pain patients. Using a randomized trial, Jamison and colleagues [58] tested the effects of combined close monitoring and cognitive behavioral treatment (education and motivational counseling) in patients at high risk for opioid misuse (due to a past history of addiction) in a pain management center. They found that no participant receiving cognitive-behavioral treatment was discharged due to aberrant behaviors, and that opioid treatment adherence and opioid misuse behaviors were better in this group than in those who did not receive the enriched treatment.

### Identification of addiction in the chronic pain patient receiving COT

Savage and colleagues introduced the four “C” criteria for identifying opioid addiction in chronic pain population: impaired *c*ontrol over drug use, *c*ompulsive use, *c*ontinued use despite harm, and unmanageable drug *c*raving [10]. However, these criteria have not been validated in clinical settings. The multiple screening and assessment tools previously identified are helpful in identification, especially if compared with scores upon admission.

**Figure 2:**
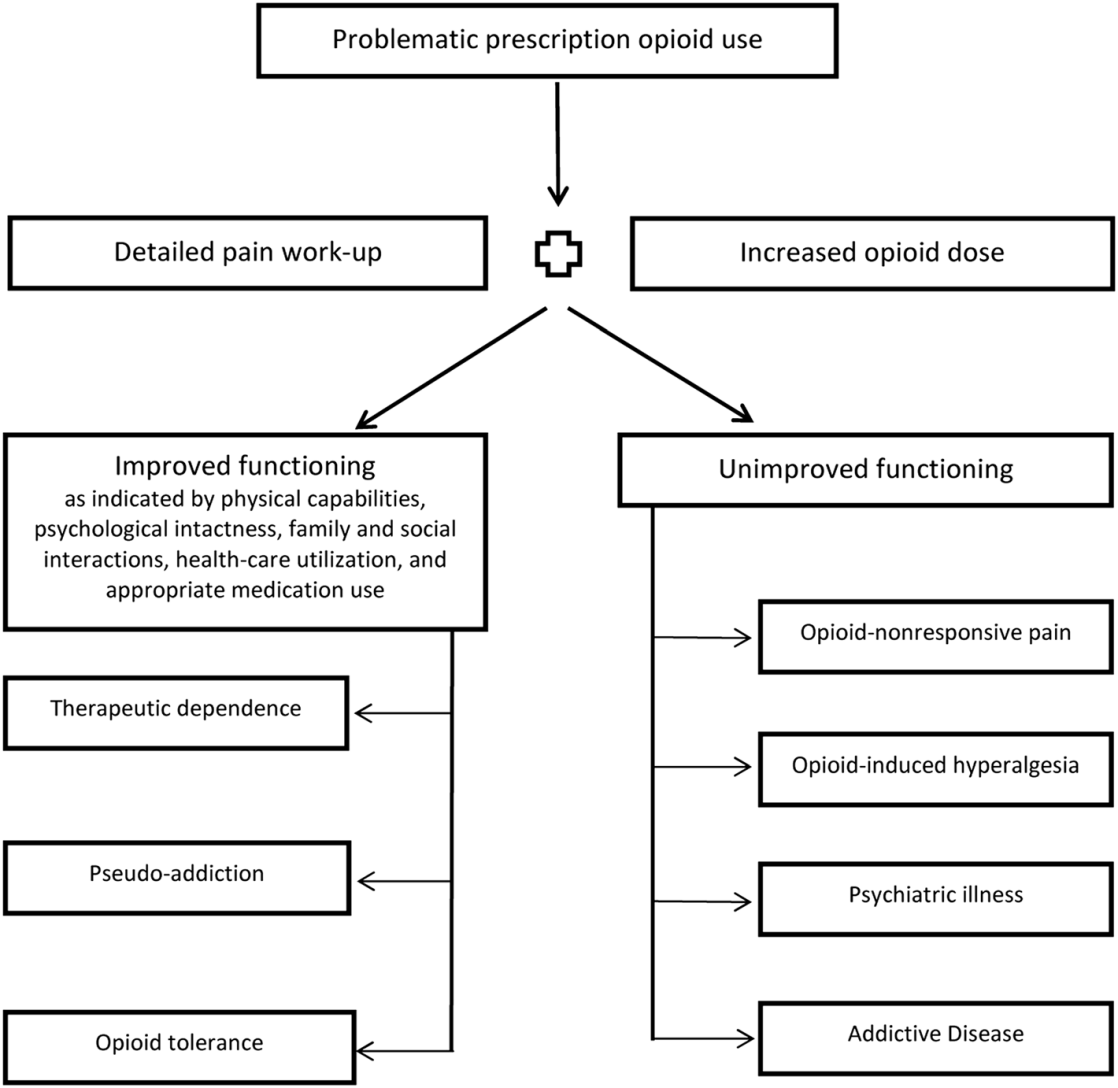
**Decision tree for interpreting aberrant prescription opioid use behavior in the chronic pain patients on opioid therapy.** Adapted from [59,60].

A strategy to distinguish between aberrant or misuse behaviors and addiction in chronic pain patients is to assess the relationship between opioid dose titration and functional restoration (Figure 2). In this approach, in response to aberrant “drug-seeking” behaviors (ie, continued complaints of pain and/or requests for more medication), the clinician increases the opioid dose in an effort to provide analgesia. Improvements in functional outcomes and quality of life, with fewer problematic behaviors, indicate that active addiction is not present. In this case, drug-seeking behaviors may reflect pseudo-addiction, therapeutic dependence, or opioid tolerance (Table 1). Effective dosing results in functional restoration.

Conversely, should overall functionality not improve with a dose increase, addiction is considered in the differential diagnosis. Listed in Figure 2 are alternate explanations for poor functional improvement, including non-opioid responsive pain; opioid-induced hyperalgesia; or an untreated psychiatric disorder. In these circumstances, clinicians should consider taper of the opioid dose and replace it with other pain-relief strategies [61] to improve function and quality of life. If the patient shows resistance to detoxification and cannot comply with the alternative treatment plan, addiction should be considered.

### Treating chronic pain in SUD patients receiving COT

#### Patients with untreated addiction: focus on addiction treatment

The authors strongly believe that patients with chronic pain and *active* addiction, regardless of type(s) of substance abused, are *not* candidates for COT [62]. Patients meeting DSM-V criteria for addiction and related disorders are, by definition, unable to achieve the goals of functional restoration. Untreated addiction results in poor functionality and, thus, will necessarily result in poor pain outcomes.

In many primary care or pain management settings, the ability to provide the comprehensive services necessary to treat patients with both pain and current addiction are sorely lacking. Patients with an active SUD should be referred to formal addiction treatment; thus, it is incumbent upon the prescribing clinician to have available a referral network of substance abuse treatment providers willing to collaborate on providing care to patients with comorbid pain and SUD. After referral, the pain clinician should continue to work closely with the SUD treatment provider to monitor use behaviors and pain outcomes.

#### Patients with addiction in remission: focus on relapse prevention

For individuals with addiction in remission, the goal of treatment is the same as that as for all chronic pain patients: to improve pain and maintain functionality. Indicators of successful pain management include the patient’s ability to comply with regimens; engage in cognitive-behavioral pain management strategies; utilize positive coping skills to manage stress; and establish better social support systems. Further, management of comorbid neuropsychiatric complications is critical to maximize functionality.

For many opioid addicts, disease remission includes opioid substitution therapy. In the context of managing pain in patients receiving methadone or buprenorphine for addiction, it is commonly assumed that the treatment opioid alone provides sufficient pain relief. Further, concerns that additional opioids put the patient at risk for untoward events, including respiratory depression and decreased level of consciousness [63], often limit opioid prescription. Although methadone and buprenorphine can be used to treat pain, their duration of analgesic action is shorter than effects on withdrawal and craving, thus dividing the daily dose and giving more frequently is the indicated strategy [64,65]. Further, patients on opioid substitution therapy develop some degree of opioid analgesic tolerance, and thus may require higher opioid doses to appreciate pain relief [40,66,67]. Studies have provided evidence that methadone maintenance patients may, in fact, have heightened pain sensitivity, and therefore have a higher opioid analgesic requirement than matched controls [68].

Regardless of the type(s) of substance previously abused, exposure to psychoactive medications can lead to relapse in patients with a recently or poorly treated SUD. Concerns of relapse may also contribute to clinicians’ reluctance to prescribe COT for patients whose addiction is in remission. The literature provides evidence that patients with successfully treated addiction can be effectively treated with opioids for chronic pain [69]. Thus, when providing COT to these patients, in addition to maximizing functionality, the treatment goals include preventing an exacerbation of the SUD.

Central to this treatment is the integration of relapse prevention strategies into the plan of care. Relapse is a predictable event in the course of addictive disease and is understood to be a process that does not occur suddenly or spontaneously and is, therefore, preventable [70,71]. The well-known social-psychology model of relapse introduced by Marlatt and Gordon [72] almost 30 years ago suggests that relapse is part of the behavioral change process and relatively common as the patient attempts to integrate new and healthier self-management behaviors into his or her life. Substance use disorders are chronic diseases for which significant behavioral change is required to successfully achieve remission.

**Figure 3:**
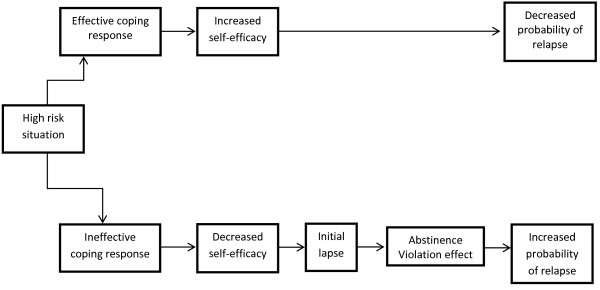
**The cognitive-behavioral model of the relapse process posits a central role for high-risk situations and for the SUD patient’s coping response to those situations.** People with effective coping responses to high-risk situations (i.e., increased self-efficacy), are at decreased probability of a relapse. Conversely, people with ineffective coping responses (decreased self-efficacy) which, together with the expectation that drug use will have a positive effect (i.e., positive outcome expectancies), can result in an initial lapse. This lapse, in turn, can result in feelings of guilt and failure (i.e., an abstinence violation effect). The abstinence violation effect, along with positive outcome expectancies, can increase the probability of a relapse. Adopted from [75].

The relapse prevention model is depicted in Figure 3. A basic assumption of the model is that relapse events are preceded by encountering a *high-risk situation*, broadly defined as “a circumstance in which an individual’s attempt to refrain from a particular behavior (ranging from any use of a substance to heavy or harmful use) is threatened” (p. 224) [73]. For patients with a history of SUD, triggers for relapse are attributed to both intrapersonal and interpersonal stressors. For patients with chronic pain, unique stressors include the losses and limitations associated with chronic pain and pain-related diminished quality of life. Although some high-risk situations (eg, negative affect, craving) seem to be universal across addictive behaviors, they vary across individuals and may change within the same individual over time [74].

Whether or not a high-risk situation results in relapse is determined by the individual's ability to engage an effective coping response to the stressor [71]. In the model, *positive outcome expectancies* and the *abstinence violation effect* are important cognitive factors in determining relapse probability. Positive-outcome expectancies refer to the anticipated positive effects of substance use (eg, getting “high,” decreasing anxiety, social rewards), which override memories of the consequences associated with use. The abstinence violation effect refers to the patient viewing a single lapse, or “slip,” as a personal failure, leading to feelings of guilt, demoralization, and hopelessness with respect to his or her ability to maintain change. More recent conceptualizations of relapse describe it as a dynamic phenomenon, and a complex nonlinear process in which various factors act jointly and interactively to influence relapse timing and severity [73].

Central to successful relapse prevention are learned cognitive and behavioral strategies the patient can employ in the face of high-risk situations. These strategies are of two broad categories: (1) a specific intervention technique designed to assist the individual in anticipating and effectively coping with high-risk situations; and (2) global self-control approaches designed to reduce relapse risk by promoting positive lifestyle changes. In that high-risk situations vary among individuals, it is critical to conduct a comprehensive assessment of substance use patterns, high-risk situations, coping skills, self-efficacy, outcome expectancies, and readiness to change, as well as to document coexisting conditions that may complicate the relapse-prevention process. To increase insight into, and self-monitoring of, problematic behaviors, the patient is encouraged to identify immediate precipitants and distal lifestyle factors related to relapse and to evaluate his or her own coping responses to high-risk situations.

Specific intervention strategies include enhancing self-efficacy by setting achievable behavioral goals and purposeful dispelling of positive outcome expectancies. With respect to global self-control strategies, patients are encouraged to incorporate stress-reduction activities into their daily life, such as exercise or meditation. The overall purpose is lifestyle balancing, which increases self-efficacy across life domains and therefore minimizes the risk of relapse.

### Relapse-prevention strategies for SUD patients receiving COT

Preventing relapse is central to effective COT in patients with SUD in remission. Clinicians must continuously assess the patient’s relative risk for it and monitor for its emergence. Further, the ability to manage a relapse episode, if one should occur, is a necessary skill of the COT prescriber. With addiction in remission, optimal functioning with appropriate opioid use can be appreciated.

#### Assessment of risk of relapse

A series of questions should be asked of the chronic pain patient regarding the status of SUD remission (Table 4). Asking these questions at each visit allows for early identification of high-risk situations and potential coping responses to these stressors.

**Table 4:** Questions to assess risk for relapse

	
•	How long has patient been in recovery?
•	How engaged is the patient in addiction recovery efforts/treatment (i.e., supportive counseling, 12-step program)?
•	What type(s) of drugs were abused?
•	What are current stressors that might precipitate relapse? These include unrelieved pain; sleep disorders; withdrawal symptoms; psychiatric symptoms, interpersonal conflicts.
•	What are current protective factors against relapse, including improved coping responses and a social support system?
•	How stable does patient feel in recovery?

#### Recognition of and monitoring for relapse

The identification of relapse in chronic pain patients receiving COT is complicated by their tendency to hide problematic use for the fear of losing access to medications. A careful monitoring plan including general and additional precautions (as described above) is critical. A relapse contract can be developed with the patient in early treatment, which is individualized to the patient and specifies steps or actions that will be taken by both the patient and clinician if relapse occurs. The patient’s behaviors with respect to the opioid-analgesic regimen provide the best evidence for the presence of active addiction. Evidence of relapse in chronic pain patients includes the presence of adverse consequences associated with opioid use, a loss of control over the use of opioids, preoccupation with obtaining opioids, and a lack of improvement in function [10].

An objective indicator of medication use is adherence to a treatment contract or medication agreement, which clearly outlines acceptable and unacceptable medication use behaviors. However, engaging in unacceptable medication-taking behaviors cannot be considered a definitive indicator of addictive disease, and rather may reflect an untreated psychiatric disorder or misunderstanding of dosing instructions. Similarly, unexpected UDT results may indicate patients’ nonadherence to opioid regimen or problematic use of medications, but it is not a specific indicator of relapse to addictive disease. Thus, clinicians should not summarily discharge SUD patients from COT based on behavioral indicators or UTD results; neither are specific to exacerbation of addiction. Rather, these findings should prompt a dialogue between the patient the clinician. Patients with a history of SUD who are nonadherent to the prescribed opioid regimen should be strongly encouraged to increase recovery efforts, and their access to opioids should be more tightly controlled. Evaluation by an addiction specialist is warranted if behaviors do not quickly resolve.

#### Management of relapse

If relapse is identified, it is critical to continue to support patients’ efforts towards recovery and maintain high levels of controls over opioid access. If attempted, opioid detoxification should be gradual so as not to elicit opioid withdrawal symptoms (usually, no more than a 20-25% dose reduction every two days). It is important not to characterize the relapse as a treatment failure but to frame it as a part of the process of recovery from an addictive disease and successful pain treatment.

Studies indicate that exposure to specific high-risk situations alone does not predict relapse, but the way in which people cope with those situations is a strong predictor of subsequent relapse or continued abstinence [76-78]. Following a relapse, a careful review of the relapse episode can be helpful. This analysis should chronicle the relapse and identify associated emotional and cognitive status that preceded it. Doing so will help the patient better recognize his/her own vulnerability to relapse as well as coping strategies that may or may not be effective.

If relapse is identified, discharging the patient from pain treatment without providing addiction intervention is not only premature, but sets the patient up for the progression of addictive disease. It is important that clinicians who prescribe COT for chronic pain are prepared with a relapse management strategy and have addiction expertise or support in place. It is critical that COT providers maintain a thoughtful and working partnership with addiction treatment providers so that pain treatment can continue while supporting addiction remission. As opposed to discharge, it is incumbent upon the pain-management practitioner to take more of an advocacy role in the management of addiction.

## Conclusion

Management of chronic pain in patients with a history of SUD with COT can be challenging, but with appropriate assessment and management, can be successful, leading to enhanced functionality and quality of life. Albeit imperfect, data suggest that up to one-quarter of chronic pain patients have an SUD history. The interrelated behavioral symptomatology of addiction and chronic pain suggests that the untreated presence of one precludes effective treatment of the other. Demographic correlates and risk factors for SUD have been well-described, and COT management is most successful when based upon risk stratification with increased control of opioid access for those classified as high risk.

The evidence is good that COT can be effective in patients with chronic pain whose SUD is in remission, suggesting that a primary goal of treatment, in addition to improving pain and maximizing functionality, is to prevent a relapse or exacerbation of addictive disease. Expanding the pain treatment plan to include specific relapse-prevention strategies and directed relapse management, if needed, is critical to appreciate the benefits of COT for patients with a history of SUD. Identifying relapse in this population can be challenging and should not be based on a single indicator. Premature discharge of the SUD patient from pain treatment provides an opportunity for addiction to worsen. It is suggested that the best chronic pain outcomes occur when the pain clinician and addiction treatment provider work in concert using a syndromal approach to treat pain and addiction.

## Competing interests

The authors declare that they have no competing interests.

## Authors’ contributions

YPC and PC collaborated on the conception of the manuscript and YPC wrote the first draft. Both authors read, edited and approved the final manuscript.
